# Supplementary material for: Prenatal Detection of Novel Compound Heterozygous Splice Site Variants of the KIAA0825 Gene in a Fetus with Postaxial Polydactyly Type A
Source: Genes (Basel). 2022 Jul 11;13(7):1230. doi: 10.3390/genes13071230 (PMC9316509; doi:10.3390/genes13071230)
Supplement: Supplementary file 1 [file genes-13-01230-s001.zip › genes-1787829-supplementary.pdf]

genes

MDPI

Online Supplementary Materials

Supplementary methods

Minigene reporter assay:

To create hybrid minigene constructs, we used the pcMINI-C vector that we developed previously, which is based on the pcDNA 3.0 mammalian expression vector and contains a multicloning site as shown in Supplementary Figure S1. We cloned DNA fragments containing a couple of exons and introns around the target variant in the *KIAA0825* gene using classical restriction and ligation methods. Primer sequences for the c.-1-2 A>T variant and the c.2247-2 A>G variant were shown in Supplementary Table 1 and Table 2, respectively.

1. Detailed steps for minigene constructs carrying the c.-1-2 A>T variant were as follows:

(1) Two pairs of nested primers, 79980-KIAA-F with 82839-KIAA-R and 80831-KIAA-F with 82478-KIAA-R, were designed. Nested PCR was carried out with normal human gDNA as the template.

(2) WT fragment amplification: the second round of nested PCR was used as the template and pcMINI-C-KIAA-KpnI-F and pcMINI-C-KIAA-BamHI-R were used as primers to amplify the 1100bp wild-type fragment of pcMINI-C.

(3) Mutant fragment amplification: pcMINI-C-KIAA-KpnI-F with KIAA-MUT-R and KIAA-MUT-F with pcMINI-C-KIAA-BamHI-R were used as primers to amplify the upstream and downstream segments, respectively. The 1:1 mixture of upstream and downstream segments was used as the template, and pcMINI-C-KIAA-KpnI-F and pcMINI-C-KIAA-BamHI-R were used as primers to amplify the 1100bp of pcMINI-C c.-1-2 A>T mutant fragment.

2. Detailed steps for minigene constructs carrying the c.2247-2 A>G variant were as follows:

(1). Two pairs of nested primers, 163815-KIAA-F and 166878-KIAA-R and 164168-KIAA-F and 166456-KIAA-R, were designed. Nested PCR was carried out with normal human gDNA as template.

(2) WT fragment amplification: the second round of nested PCR was used as the template, and pcMINI-C-KIAA-c.2247-EcoRI-F and pcMINI-C-KIAA-c.2247-NotI-R were used as primers to amplify the 1100bp wild-type fragment of pcMINI-C.

(3). Mutant fragment amplification: PcMINI-C-KIAA-c.2247-EcoRI-F with KIAA-MUT-R and KIAA-MUT-F with pcMINI-C-KIAA-c.2247-NotI-R were used as primers to amplify the upstream and downstream segments, respectively. The 1:1 mixture of upstream and downstream segments was used as the template, and pcMINI-C-KIAA-c.2247-EcoRI-F and pcMINI-C-KIAA-c.2247-NotI-R were used as primers to amplify the 1035bp of pcMINI-C c.2247-2 A>G mutant fragment.

3. The hybrid minigenes were confirmed by sequencing and transfected into HEK293T and HeLa cells using Lipofectamine® 2000 (Thermo Fisher Scientific, Waltham, MA, CA). After 48 hours, total RNA was extracted using the RNA extraction kit (Trizol RNAiso PLUS, 9109, TaKaRa), retro-transcribed with the Superscript III reverse transcriptase (HifairTM 1st Strand cDNA Synthesis SuperMix for qPCR(gDNA digester plus), 11123ES70, YEASEN) and the resulting cDNA was PCR-amplified. The amplified products were analyzed on 1.5% agarose gel electrophoresis and subsequently sequenced by Sanger sequencing.

Supplemental Figures

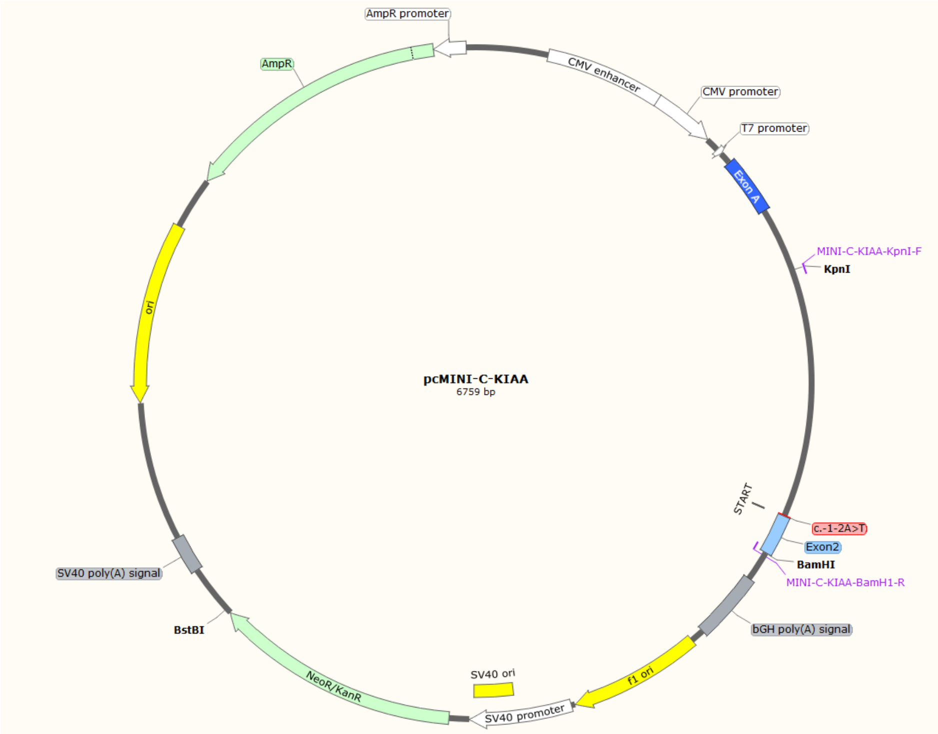

（a）The pcMINI-C vector construction map for the minigene assay of c.-1-2 A>T variant.

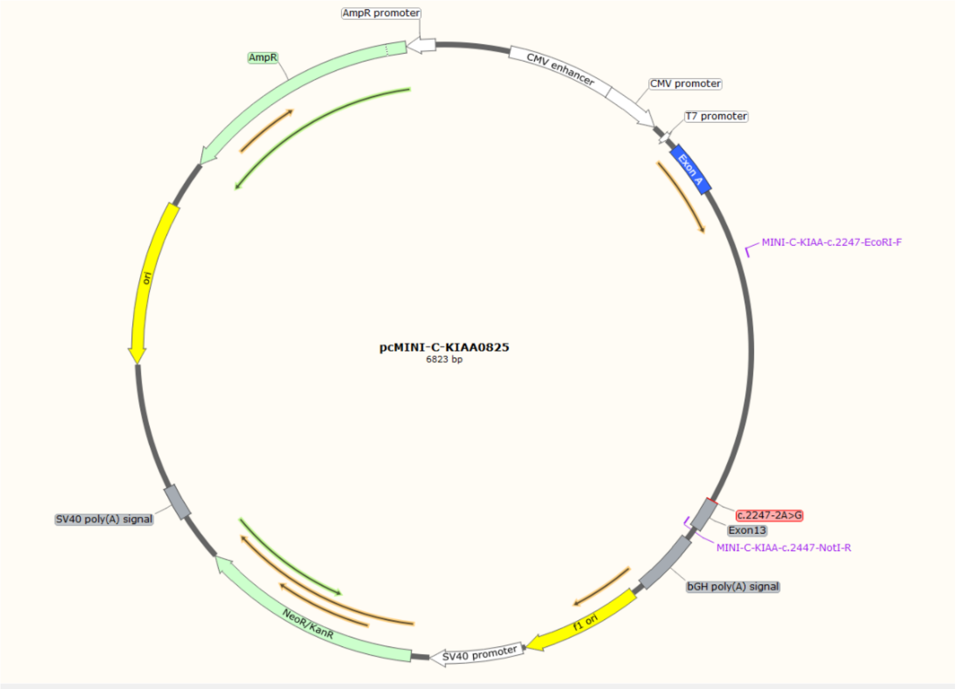

（b）The pcMINI-C vector construction map for the minigene assay of c.2247-2 A>G variant.

Figure S1. The pcMINI-C vector construction map for the minigene assay.

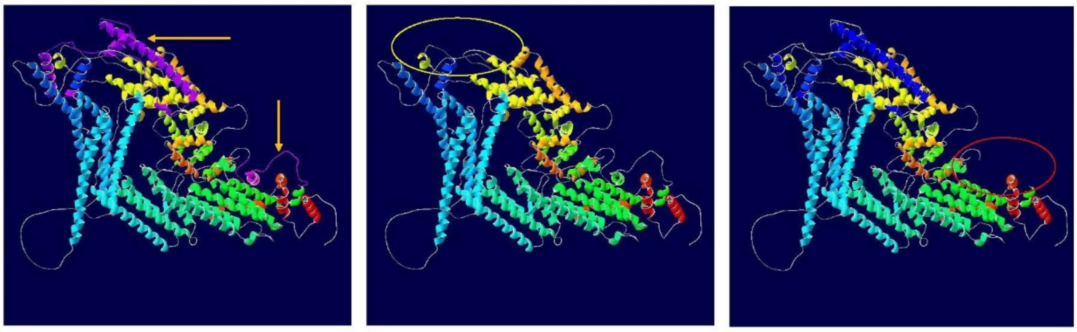

(a) Wild type (b) p.Asn1_Glu112del (c) p.Phe751_Thr787del

Figure S2. The pcMINI-C vector construction map for the minigene assay.

AF-Q8IV33-F1-model_v2 was computed using the AlphaFold Protein Structure Database. Protein structures of wild and mutant types were produced by SwissPdb Viewer.

References

[1] Guex, N. and Peitsch, M.C. (1997). SWISS-MODEL and the Swiss-PdbViewer: An environment for comparative protein modeling.

[2] Electrophoresis 18, 2714-2723.Jumper, J et al. Highly accurate protein structure prediction with AlphaFold. Nature (2021).

[3] Varadi, M et al. AlphaFold Protein Structure Database: massively expanding the structural coverage of protein-sequence space with high-accuracy models. Nucleic Acids Research (2021).

Supplementary Tables

Table S1: The number of variants after each filtering step.

Filtering step	Variant number Fetus
Initial	114,573
1. Exclude the variants outside exonic and splicing regions	36,549
2. Exclude the variants with MAF* > 0.01	3,049
3. Exclude the synonymous variants in exome	1,936
4. Include the variants inherited in de novo, AR and XR patterns#	315 variants in 105 genes

*MAF: Minor allele frequency in gnomAD, 1000 genome and Exome Aggregation Consortium database
AR, autosomal recessive; XR, X-linked recessive.

Table S2: Primer sequences in the minigene constructs carrying the c.-1-2 A>T variant.

Primer name	Primer sequence (5′-3′)
79980-KIAA-F	aatggtagtggcagaaaggc
80831-KIAA-F	ctcacactctgtccttgctc
82478-KIAA-R	gagaggatttctgggatggg
82839-KIAA-R	gttgcctgttgcaatttgca
pcMINI-C-KIAA-KpnI-F	ggtaggtaccaggggtgctagagaaactca
PcMINI-C-KIAA-BamHI-R	tagtggatcccttgcagcattttgttcaat
KIAA-MUT-F	ttattcttgctcctctgaatggattgggatg
KIAA-MUT-R	catcccaatccattcagaggagcaagaataa

Table S3: Primer sequences in the minigene constructs carrying the c.2247-2 A>G variant.

Primer name	Primer sequence (5′-3′)
163815-KIAA-F	aacctgctctcctcactact
164168-KIAA-F	ggggagagaagggtcagatt
166456-KIAA-R	ggtccttgaagatggggtct
166878-KIAA-R	ggttgatccgagcaagcttg
KIAA-MUT-F	tatttgtatttccctgggacttttcagcatg
KIAA-MUT-R	catgctgaaaagtcccagggaaatacaaata
pcMINI-C-KIAA-c.2247-EcoRI-F	ggtggaattcgccctctctagatccttagg
pcMINI-C-KIAA-c.2247-NotI-R	tcgagcggccgcgctgagtaaagaagggtagaa
